# Multitasking Effects on Perception and Memory in Older Adults

**DOI:** 10.3390/vision6030048

**Published:** 2022-08-04

**Authors:** Giulio Contemori, Maria Silvia Saccani, Mario Bonato

**Affiliations:** 1 Department of General Psychology, University of Padova, 35131 Padova, Italy; 2 Padova Neuroscience Center, University of Padova, 35131 Padova, Italy

**Keywords:** memory, aging, dual-task, cognitive load, cost

## Abstract

Performing multiple tasks in parallel is detrimental to performance, a phenomenon generically referred to as dual-task interference (DTi). Several variables can modulate DTi at the individual level, and increasing age is typically described as negatively affecting response costs. In this study, we investigated, in 252 healthy adults aged between 50 and 89 years, how age modulates the detrimental effect of DTi during the encoding of images. We combined a visual memory task and a sustained attention task (i.e., an auditory version of the continuous performance task, ACPT) in three separate blocks. In the first block, participants had to perform a four-alternative forced-choice recognition of previously memorized images without having attended to ACPT sounds during the encoding. In the second block, during memorization, participants had to press a response key when detecting the letter “X” within a stream of letters (Low Load). In the third block, they had to respond only when the letter “X” was preceded by the letter “A” (High Load). The results showed that overall performance linearly decreased with age. In contrast with our predictions, DTi was stable across different ages. Finally, using a cluster-based approach, we found that participants who paid the highest costs when dual-tasking also demonstrated, on a self-administered cognitive screening significantly lower scores than peers. These new types of tests, which ask for concurrent task performance, might become useful for detecting outlier performance that might anticipate or correlate with aging–related cognitive decline.

## 1. Introduction

In laboratory settings, our attention is completely focused on the task at hand, and visual processing is almost invariably studied in isolation. In ecological situations, the environment is much more articulated and the contextual information more complex. When processing, for instance, new visual information, the context can strongly influence the later recalling of the object’s identity, a process commonly referred to as contextual binding [1]. Part of the complexity of everyday situations is that multiple sources of information need to be concurrently processed. As a consequence, it seems unclear to what extent results obtained in a lab generalize to everyday perception and attention [2], as outside the lab parallel processing is the rule rather than the exception. Dual-tasking, and its assessment, captures the interaction of different executive functions and therefore mimics the requirements of everyday life, thus overcoming the trade-off between valid and ecological settings.

In this study, we analyzed the ability to encode and retrieve visual information with and without divided attention in a group consisting of middle-aged and older healthy adults (50–89 years old). Dividing one’s attention to simultaneously process two (or more) items can drastically hamper performance, a phenomenon generically called dual-task interference (DTi) [3]. DTi occurs when simultaneous execution causes deterioration in the performance of one or both tasks. Over the last few decades, an ever-growing field of research in cognitive and experimental psychology has tried to uncover the mechanisms involved in dual- and multi-tasking. The three most influential “attentional” theories trying to explain the DTi in humans are: (1) the central capacity sharing model, postulating that resources must be re-distributed between the tasks [4]; (2) the bottleneck model, suggesting that when two different tasks are processed simultaneously, the central stage of response selection can only process one task at a time, and the tasks have to be completed sequentially [5]; and (3) the cross-talk model, proposing that when stimulus and response processing share parts of the same neural network across the two tasks, the interference among central operations originates the cost [6,7]. Regardless of the differences between the models, a general characteristic of multi-tasking is that it brings the cognitive processing system to its limits and results in performance deterioration. These limits may manifest either as difficulty in monitoring the two tasks (executive functions limit) or more quantitatively: e.g., difficulty in adequately processing all the information (working memory capacity limit) [8,9].

Dual-task paradigms almost always involve a primary task performed in isolation or associated with a secondary (resource-depleting) task. The nature of the task(s) can be cognitive or motor, and the degree of interference resulting from their simultaneous execution depends greatly on their combination. When both tasks are cognitive, they can either test the same domain (e.g., two concurrent working memory tests) or two different domains. Motor tasks often assess an aspect of walking, but other examples also exist (for reviews about the dual-task taxonomy see [10,11,12]).

Dual-tasking paradigms have also been increasingly used in clinical settings. They provide diagnostic capability for older people and can be used for preventive or rehabilitative training [13]. In particular, cognitive–motor dual tasks have been proven sufficiently sensitive to detect pathological cognitive impairment in the elderly [14]. In contrast, cognitive–cognitive dual tasks are not so widely employed for clinical purposes, even though in clinical settings, data on performance in computerized tests could be easily collected, analysed and stored [15].

The present study investigates how age impacts cognitive–cognitive dual-task interference by using a newly designed experimental paradigm. We combined a visual memory (primary) task with a sustained attention (secondary) task. The memory task consisted of a series of images to be memorized, followed by forced-choice recognition. The sustained attention task was an auditory version of the continuous performance task (ACPT), two versions of which were designed to manipulate cognitive load through different instructions between blocks and was specifically revised for online self-administration [16,17]. The continuous performance task is a Go-No-go test developed by Rosvold, Mirsky, Sarason, Bransome, and Beck in 1956 [18] to measure sustained attention and executive functions [19,20,21,22]. By designing this task, we wanted to generate visuo-auditory DTi in an early phase (i.e., during the visual encoding), hypothesizing a later decrease of mnestic performance in the forced-choice recognition. Previous research has shown that when DTi is applied during encoding in recognition tasks, voluntary control is reduced, and the implementation of coping strategies is prevented [23,24,25]. Previous studies also suggest that recognition tasks are supported by two types of largely independent cognitive processes: recollection and familiarity [26,27]. Familiarity is a more automatic form of recognition that relies on the different degrees characterizing the feeling of “having already experienced” something. Recollection is a more controlled process and generally involves the retrieval of contextual details [28]. While, broadly speaking, recognition abilities decline with age, familiarity-based memory has been described as being relatively spared with respect to recollection-based memory during normal aging [29].

In a previous study from our group [30], we coupled an in-person computer-based visual memory task with a phonemic fluency task performed during the encoding phase of images in a group of healthy adults (age range 50–77 years). When the response consisted of a forced-choice test, older adults suffered more than younger ones from the increased cognitive load. This pattern, however, was simply due to young participants performing at ceiling in the easy, single-task condition. Moreover, in the free recall test, the same interaction between cost and age was not significant. It is therefore likely that in that study, the forced-choice condition was too easy for the younger participants, particularly in the single-task condition.

Capitalizing on these preliminary findings, in the present study, besides changing the type of concurrent load we modified several aspects of the experimental paradigm to reliably prevent ceiling and floor effects. To avoid possible ceiling effects in the younger participants, we increased the number of images to remember. To avoid possible floor effects in older participants, we discarded the free recall response modality, and we maintained only the four-alternative forced-choice procedure with targets and foils of the same category. Images were selected from a dataset that ensured a high degree of similarity between targets and foils so that the contribution of familiarity was favored over that of recollection [31]. DTi is known to heavily disrupt recollection-based recognition memory while largely sparing familiarity-based recognition memory [25]; splitting attention with DTi impacts conscious recollection by disrupting the encoding and organization of to-be-remembered material [23,24,25].

Maximizing the potential contribution of familiarity to recollection also results in a second aspect relevant to the study. Although the literature on this topic is controversial, according to some studies elderly people with mild cognitive impairment (MCI) or early Alzheimer’s disease tend to perform significantly worse than healthy peers in tasks specifically designed to test familiarity [32,33]. On the contrary, in tasks designed to test recollection, this difference tends to be less prominent [32,33]. Therefore, familiarity-based tasks qualify as potential candidates for the early detection of cognitive impairments [34,35]. In our task, we expect an older person with a familiarity deficit to show greater interference than a peer with no such impairment. Such a drop in performance could be an indicator of the presence of other subtle cognitive deficits, potentially anticipating cognitive decline. It is therefore interesting to observe how the occurrence of DTi varies not only with age but also between individuals of the same age. To this aim, given the protective effect that education has against cognitive decline [36], we also investigated the net effect of schooling on DTi. In the Italian population, schooling and age are expected to correlate with an R^2^ close to 40 [37]. Despite this strong correlation, education could have an independent effect from that of age and might potentially mitigate its effects.

### Hypothesis

In the present study, we investigated how age impacts cognitive–cognitive DTi between a visual memory (primary) task and a sustained attention (secondary) task. The memory task consisted of a set of images to be memorized before responding to the forced-choice recognition task. As a concurrent, resource-depleting task, we used an auditory version of a continuous performance task (ACPT). It allowed manipulating load levels in different conditions (No Load vs. Low Load vs. High Load) by using stimuli in a different (auditory) modality, therefore minimizing structural interference due to stimuli overload in the same modality.

We expected a negative impact of age and dual-task difficulty on mnestic performance. Furthermore, we expected a positive relationship between age and the amount of DTi in both the memory and sustained attention tasks. Because previous literature has shown that, as opposed to mnestic tasks, in sustained attention tasks older people generally perform comparably to younger people, at least when there is no time pressure [38], we expected a more pronounced effect of DTi on the memory task than on the ACPT. We also expected an inverse correlation between DTi and education.

Regarding individual performance, we predicted that we would find a trade-off between the cost in the memory task and the cost in the ACPT due to individual preferences in task prioritization. Finally, under the hypothesis that cost in a dual task may be an indication of subclinical cognitive impairment, we clustered participants into three groups based on individual DTi and investigated whether the group with the highest total cost was characterized also by the lowest cognitive scores.

## 2. Materials and Methods

### 2.1. Participants

A total of 340 Italian-speaking volunteers aged between 50 and 89 years participated in the study. Participants were recruited by members of the research laboratory by word of mouth and were unaware of the hypotheses and aims of the study. Each participant received an e-mail containing general instructions and a link to participate in the study.

The inclusion criteria were normal or corrected-to-normal vision and hearing. The exclusion criteria were the presence of psychiatric disorders, a history of addiction disorders (alcohol or drug abuse), and the presence of severe neurological disorders (MCI or dementia).

**Figure 1:**
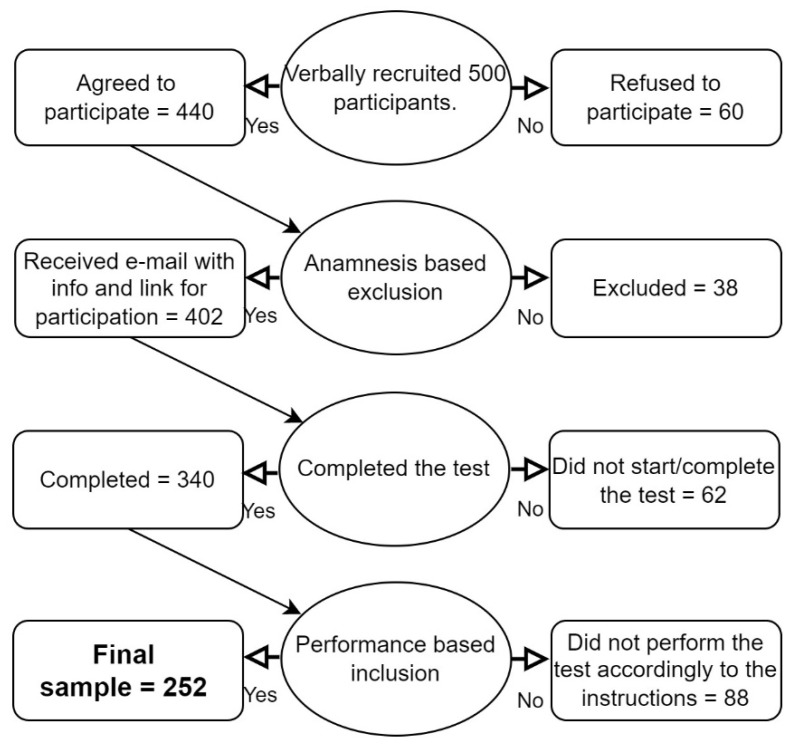
Diagram of participants’ recruitment.

To include in the further analysis only those compliant participants who carefully followed the instructions, we defined two additional inclusion criteria based on ACPT performance. The first criterion consisted of having answered, for each ACPT block, to at least one of the (three) target letters presented. The second criterion was to present less than 50% false alarms in at least one of the two ACPT blocks. To be included in the analysis, participants had to fulfill both criteria. The combination of these two criteria made it possible to exclude all those participants who had misunderstood the ACPT instructions in one of the two blocks and for whom there was no certainty that load modulation had taken place. In particular, the second criterion was designed to remove those participants who, in the High Load condition, responded systematically even to stimuli that were not the target sequence, de facto nullifying the load difference between the “X” and “AX” versions of the ACPT. Eighty-eight participants did not meet the above-described criteria and were removed from subsequent analyses. A schematic representation of the recruitment process, including the post-task exclusions, is shown in Figure 1.

**Figure 2:**
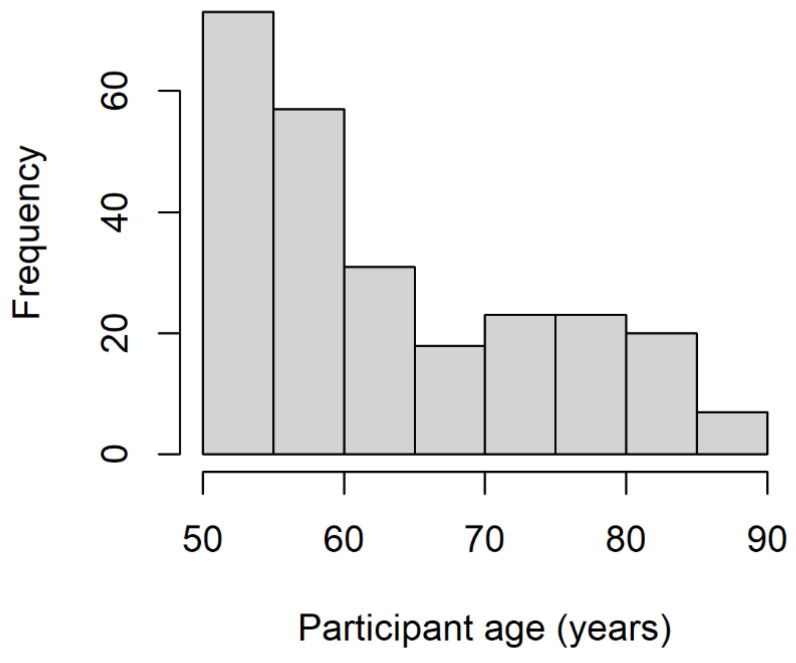
Frequency plot of the participants’ ages. Each bar represents the number of participants in each age group (5 years).

The final sample encompassed 252 participants (145 females) with a mean age of 63.8 years (SD = 10.7). The participants’ age distribution is shown in Figure 2. All participants gave explicit informed consent through an online form prior to their inclusion in the experiment and received no compensation for their participation. The experimental protocol was approved by the Ethics Committee for Psychological research of the University of Padua.

### 2.2. Software and Materials

We built the experimental protocol using HTML (HyperText Markup Language), CSS (Cascading Style Sheets), and jsPsych, an open-source JavaScript library developed specifically as a framework for web-based psychological experiments [39]. We extended jsPsych’s capabilities using the “jspsych-psychophysics” plugin which allowed for very accurate timing of visual and auditory stimuli [40]. Through this methodology, it is possible to remotely measure accuracy and reaction times for audio-visual stimuli with laboratory precision [41,42,43]. jsPsych is a front-end JavaScript library that runs entirely on the participant’s computer. To make the protocol available through the internet, we uploaded the experimental material to a web server with a JATOS instance installed [44]. JATOS is an open-source, cross-platform web application for the management of data and links to the experiment. Each participant received the same access link. The link allowed only one access for each participant based on the IP address of their computer. The web server hosting the experiment and the associated secure database were located at the Department of General Psychology of the University of Padua.

To take part in the study, participants needed a computer with internet access, a mouse, a keyboard, and audio speakers. To avoid unforeseen issues with the rendering of the experimental webpage, we limited the execution of the experiment to three browsers (Chrome, Firefox, and Edge) which were intensively tested by the experimenters beforehand.

### 2.3. Experimental Design

The experiment consisted of a self-administered online protocol composed of a cognitive screening and three experimental tasks. A detailed description of the experiment was provided to each participant via e-mail along with the indication to perform the task while seated at a table in a sufficiently illuminated, silent, and comfortable environment. If the participant was not sufficiently familiar with the computer, a caregiver was required to help preparing the set-up. The order of the tests was kept constant, and the participants were informed that they could interrupt the experimental procedure at any time and withdraw their consent to the use of their data. The caregiver was recommended not to help participants during the experiment.

### 2.4. Education Level

Prior to the cognitive screening, participants had to fill out an education-level assessment form. In the first drop-down menu, participants had to select their last obtained degree/diploma. Participants then had to indicate the total length of other educational or vocational courses attended since their last degree/diploma. Courses lasting less than 6 months were not considered. Based on the total duration of formal education, we then calculated the Education score, an index of individual education independent of age. To calculate the Education score, we used the method proposed by Nucci et al. in 2012 [37,45] and described in the data analysis (see Section 3).

### 2.5. Cognitive Screening

The cognitive screening was performed with the auto-Global Examination of Mental State, a newly devised instrument for self-administered online cognitive screening. The auto-GEMS is a computerized adaptation of the tele-GEMS, a battery designed for remote administration via telephone (Available online: https://osf.io/r3ta5/ accessed on 20 June 2022), and which is itself a derivative of GEMS, a recently published paper-and-pencil screening [46]. The auto-GEMS test set has been adapted for self-administration, whereby each participant responds autonomously by using a mouse and a keyboard, and the final scoring is automatic. It consists of a combination of closed, multiple-choice questions and structured tests. The domains tested are spatial and temporal orientation, short and long-term memory, spatial skills, verbal skills, and executive functions.

### 2.6. Experimental Paradigm

The experimental paradigm was composed of three blocks: one single-task block and two dual-task blocks. The dual-task blocks included a visual memory task (primary) and a sustained attention task (secondary). The memory task was a four-alternative forced-choice recognition task (4AFC) consisting of an encoding phase followed by a response phase, while the sustained attention task was an auditory version of the continuous performance task (ACPT).

**Figure 3:**
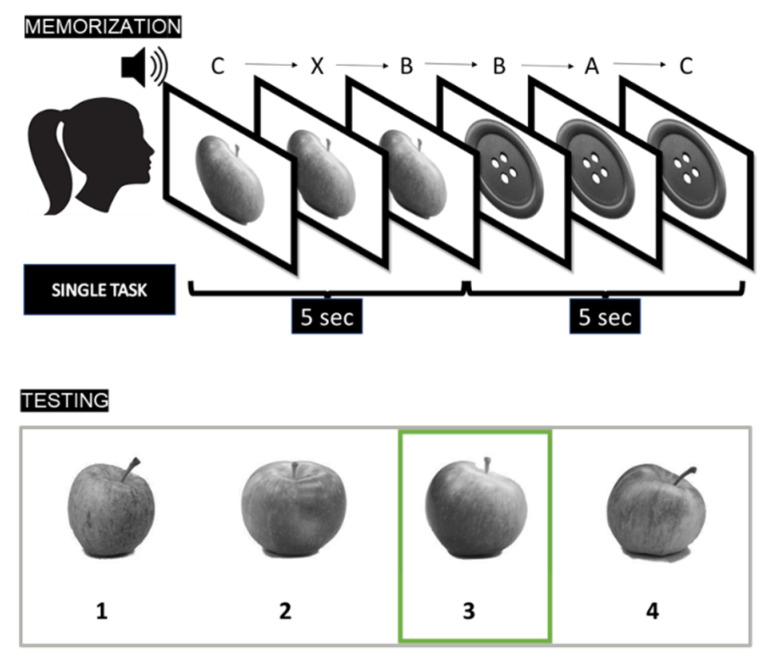
A representative sequence of the memory task. During the encoding phase (**upper panel**) 15 images were shown in sequence for 5 s each. Concurrently, a stream of 45 auditory stimuli (letters) was played with an onset asynchrony of 1666 ms. In the No-Load condition (NL), participants had to ignore the auditorily presented letters. In the Low-Load condition (LL), participants had to press a response key each time the letter “X” was presented. In the High-Load condition (HL), participants had to press a response key whenever the letter “X” was preceded by the letter “A”. During the testing phase (**lower panel**), a forced-choice image recognition was performed. One of the four images consisted of a previously presented (target) image (here image number 3, highlighted in green).

In all blocks, during the encoding phase participants were presented with a stream of images and an audio stream of letters. While in the first block participants had to ignore the letters (no-load), in the second (low-load, i.e., LL) and third block (high-load, i.e., HL), they had to respond to “A” or to “AX”, respectively. Figure 3 shows a schematic representation of the task.

#### 2.6.1. Memory Task

During the encoding phase, participants were asked to memorize 15 sequentially presented black and white images representing inanimate objects. Participants were informed they would be later asked to recognize the correct stimulus among four alternatives. Images were randomly shuffled between participants and counterbalanced across conditions. All images were selected from a database specifically created for memory studies that contained 50 different target categories. Every target image was presented in the center of the screen for 5 s, in a continuous stream.

In the recognition phase, four different images belonging to the same object category (e.g., an apple, see Figure 2) were simultaneously presented. For each target image, we selected three foils: one with a high, one with an average, and one with a low degree of similarity with the target. This way, the degree of similarity between the target and the foils was kept constant within each trial. This was possible because, within each category, multiple similar images were available together with a quantitative estimate of the similarity between images [31,47]. The order of the recognition trials and the position of the target among the four alternatives were randomized. In the recognition phase, participants were explicitly asked to select the most plausible stimulus (with no time limit). The first block was preceded by a short practice session (with three target items). To avoid familiarization with the stimuli, each target category was presented only once.

#### 2.6.2. Auditory Continuous Performance Task (ACPT)

In the LL and HL conditions, participants had to perform the ACPT while memorizing the images. They had to monitor a series of auditory stimuli (letters A, B, C, D, and X) and press the spacebar whenever the target letter was presented (see Figure 2). In each block, there were 45 auditory stimuli separated by a stimulus onset asynchrony of 1666 ms. During each image presentation (memorization phase), three different auditory stimuli were presented. There were three target letters for each block. In the LL block, participants were instructed to respond (via key press) whenever the target stimulus “X” was presented (ACPT-X). In the HL block, participants were instructed to respond (via key press) whenever the target sequence “AX” was presented (ACPT-AX).

## 3. Data Analysis

Data analysis was performed using R [48]. Data were analyzed by means of a generalized logit-linear mixed model for binomially distributed outcomes (GLMM). GLMMs are particularly suitable for analyzing complex datasets with repeated or grouped observations and are virtually unaffected by unbalanced datasets, such as participants’ ages in our case [49,50].

We performed an omnibus test based on the type II Wald chi-square test with the “Anova” function from the “CAR” package [51]. When necessary, Benjamini–Hochberg corrected post-hoc comparisons were performed by means of the “glht” function from the “lsmeans” package [52]. We included Cognitive Load (No Load vs. LL vs. HL) as a within-subject factor and Age as a between–subject continuous variable. Participants and Cognitive Load were entered as random effects in the model. By doing so, we assumed a by-subject variation in the intercept for each memory score condition.

A similar omnibus test was performed for the ACPT score and for the Education score. The Education score was calculated by removing the influence of age from the individual duration of schooling. We followed the approach originally proposed by Nucci et al. (2012) for the calculation of the CRI-Education. First, we regressed out the age effect from the raw years of education; we then standardized and transposed the residuals to a new scale (mean = 100, SD = 15) [37].

To check for patterns in the allocation of resources among the two tasks, we also included a Pearson’s correlation between the cost in the ACPT and in the memory task.

The cost was calculated by subtracting the values of the dual-task blocks from the baseline block, whereby positive values index performance deterioration/DTi. In absence of the single-task baseline condition, the cost in the ACPT was calculated by subtracting the performance obtained in the HL from the one obtained in the LL (LL–HL). For the memory task, we calculated three different costs/DTi, one for each possible combination of the load levels: No Load–LL, Load–HL, and LL–HL. Positive cost values, therefore, indexed an effect in the expected direction, i.e., the No Load block performance being better than that in the LL block and the LL performance being better than the HL performance.

Finally, to test for the presence of subgroups of participants with different cognitive profiles, we included a cluster analysis based on the k-medoids algorithm carried out with the “cluster” package in R. The clustering was done by calculating Euclidean distances (root sum-of-squares of differences) between the two cost indices from the memory test (No Load minus LL or HL for memory DTi) and the cost index from the ACPT (LL–HL for ACPT DTi). Next, we tested for group differences in age and auto-GEMS score between the three clusters by means of a Kruskal–Wallis rank sum test. Pairwise comparisons were performed by means of (three) one-tailed Wilcoxon tests for unpaired samples with Benjamini–Hochberg correction for multiple comparisons.

## 4. Results

We report below the results for the memory task, the ACPT, the cost correlations, and the cluster-based analysis, in that order.

### 4.1. Memory Task (Primary)

Descriptive statistics for the memory task are reported in Table 1.

**Figure 4:**
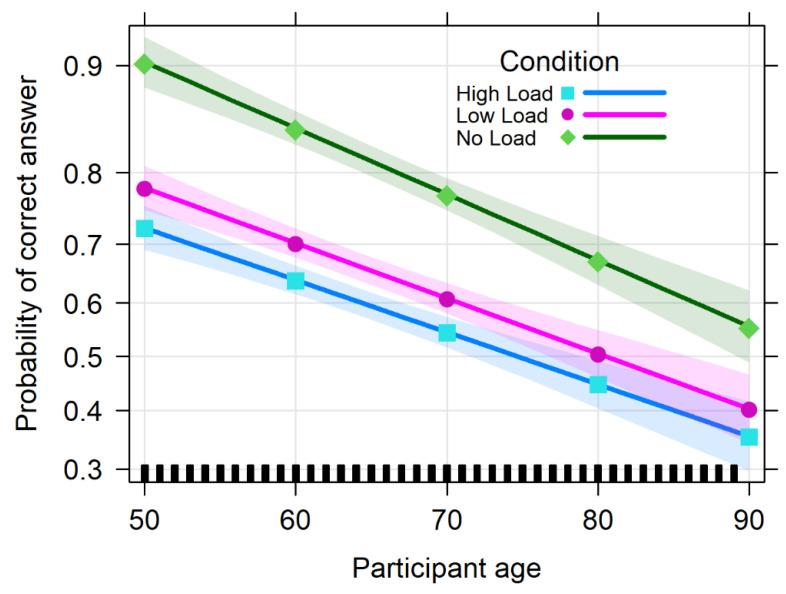
Performance in the memory task (memory score) as a function of Age (in years), reported separately for the three Cognitive Load conditions (No Load, LL, and HL). Bands represent 95% confidence intervals.

The analysis of deviance with the type II Wald chi-square tests for the memory score showed a significant effect of Cognitive Load (Chisq = 342.867, df = 2, *p* < 0.001) with the No Load condition resulting in a higher score with respect to both the LL (0.841, se = 0.0619, z = 13.592, *p* < 0.001) and HL conditions (1.134, se = 0.0620, z = 18.288, *p* < 0.001). Performance in the LL condition also was significantly better than in the HL condition (0.292, se = 0.0542, z = 5.394, *p* < 0.001). We found a significant effect of Age (Chisq = 112.918, df = 1, *p* < 0.001), with the score decreasing with greater participant age. Crucially, the interaction between Cognitive Load and Age was not significant (Chisq = 5.223, df = 2, *p* = 0.073), meaning that the age-related decrease in performance was equivalent across load conditions. Results for the Wald chi-square tests are summarized in Table 2. The performance pattern for the memory task is shown in Figure 4.

### 4.2. Auditory Continuous Performance Test (ACPT-X and -AX; Secondary Task)

Descriptive statistics for the ACPT task are reported in Table 3.

**Figure 5:**
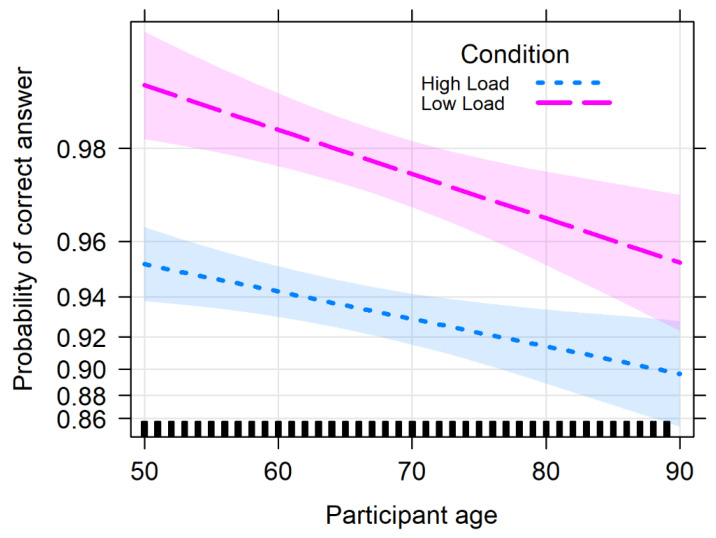
The ACPT score as a function of Age and Cognitive Load (ACPT-A: Low Load, ACPT-AX: High Load). Bands represent 95% confidence intervals.

The analysis of deviance with the type II Wald chi-square tests for the ACPT score showed a significant effect of Cognitive Load (Chisq = 95.524, df = 1, *p* < 0.001), with the LL condition resulting in a higher score than the HL condition (1.2, se = 0.123, z = 9.723, *p* < 0.001). We found a significant, detrimental effect of Age (Chisq = 12.835, df = 1, *p* < 0.001), while the interaction between Cognitive Load and Age was not significant (Chisq = 1.603, df = 1, *p* = 0.206), resembling the pattern already described for the memory task (see Figure 5). Results for the Wald chi-square tests are summarized in Table 4.

### 4.3. School Education

Descriptive statistics for Age, years of schooling, and Education scores are reported in Table 5.

**Figure A2:**
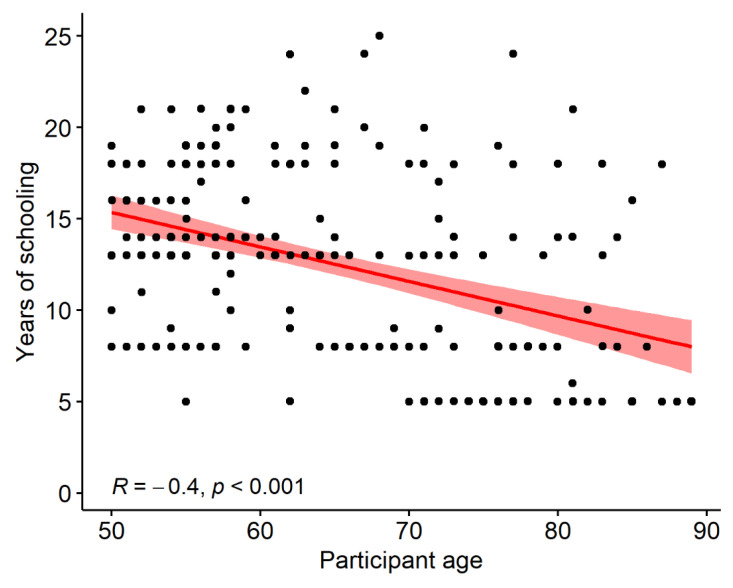
Years of schooling as a function of age.

As expected, there was a strong, significant negative correlation between Age and years of schooling (R= −0.405, t = −7.003, df = 250, *p* < 0.001). Notably, the intercept (y = 24.782) and the angular coefficient (−0.189x) were comparable to those found in a larger Italian sample by Nucci at al. (2012) (y = 21.169 − 0.1642x) [37]. The correlation plot depicting the years of schooling as a function of age is shown in Figure A2 (see Appendix B).

The analysis of deviance with the type II Wald chi-square tests for the memory score showed a significant effect of Cognitive Load (Chisq = 340.731, df = 2, *p* < 0.001), with the No Load condition resulting in a higher score with respect to both the LL (0.845, se = 0.0620, z = 13.638, *p* < 0.001) and HL conditions (1.137, se = 0.0625, z = 18.181, *p* < 0.001). Performance in the LL condition also was significantly better than in the HL condition (0.292, se = 0.0542, z = 5.390, *p* < 0.001). We also found a significant effect of Education score (Chisq = 6.298, df = 1, *p* = 0.0121), with memory scores increasing as the Education score increased. The interaction between Cognitive Load and Education score was not significant (Chisq = 1.603, df = 2, *p* = 0.558). Results for the Wald chi-square tests are summarized in Table 6.

### 4.4. Cost & Correlations

The cost in the memory task was calculated by subtracting the score in the LL or HL condition from the score in the No Load condition. Both differences were significantly different from zero (No Load–LL memory DTi = 0.145, t = 14.257, df = 251, *p* < 0.001; No Load–HL memory DTi = 0.201, t = 19.31, df = 251, *p* < 0.001), indicating a deterioration of performance in the expected direction, namely, worse performance under load. Also, the cost calculated by subtracting the LL from the HL was significantly different from zero (LL–HL memory DTi = 0.0561, t = 5.027, df = 251, *p* < 0.001), indicating the expected difference between an easier and a more difficult load condition.

**Figure 6:**
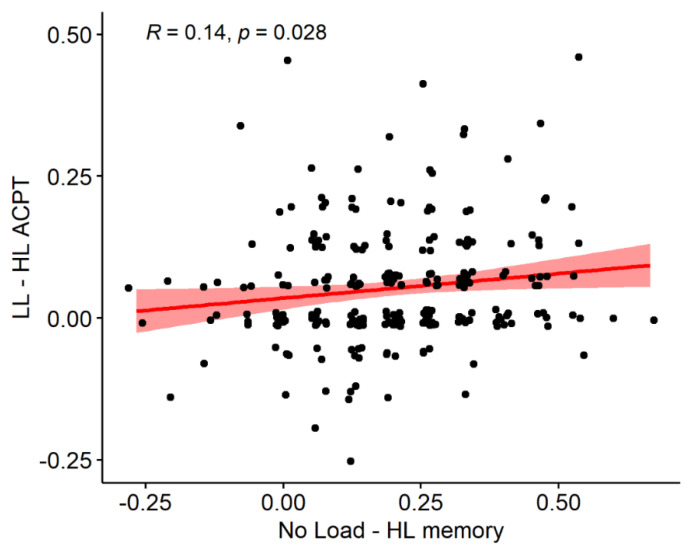
Correlation between the mnestic dual-task interference DTi (No Load–HL) and ACPT DTi (LL–HL). The bands represent 95% confidence intervals. Positive values indicate that more errors were committed in the High Load condition than in the Low Load (ACPT) or No Load (memory task) conditions.

The cost in the ACPT also significantly differed from zero (LL–HL ACPT DTi = 0.0526, t = 8.069, df = 251, *p* < 0.001). We found significant correlations between measures of dual-task costs both when comparing performance indexes derived only from the memory task and when comparing mnestic cost with ACPT cost. More specifically, concerning the memory task costs, we found a significant positive relationship between the No Load–LL memory DTi and the No Load–HL memory Dti (R= 0.414, t = 7.196, df = 250, *p* < 0.001). Concerning the comparisons across tasks, we found a significant positive correlation between the No Load–HL DTi in the memory task and the LL–HL DTi in the ACPT (R= 0.138, t = 2.211, df = 250, *p* = 0.028), as shown in Figure 6. However, we found no correlation between the No Load–LL memory DTi and the LL–HL ACPT DTi (R= 0.104, t = 1.655, df = 250, *p* = 0.099), and no correlation between the LL–HL memory DTi and the LL–HL ACPT DTi (R= 0.034, t = 0.543, df = 250, *p* = 0.587).

No significant correlation was found between the No Load–HL memory DTi and the Education score (R= −0.0741, t = −1.175, df = 250, *p* = 0.241) or between the No Load–LL memory DTi and the Education score (R= −0.107, t = −1.694, df = 250, *p* = 0.0914). On the contrary, a significant negative correlation was found between ACPT cost and the Education score (R= −0.14, t = −2.167, df = 250, *p* = 0.0312).

### 4.5. Cluster Analysis

**Figure 7:**
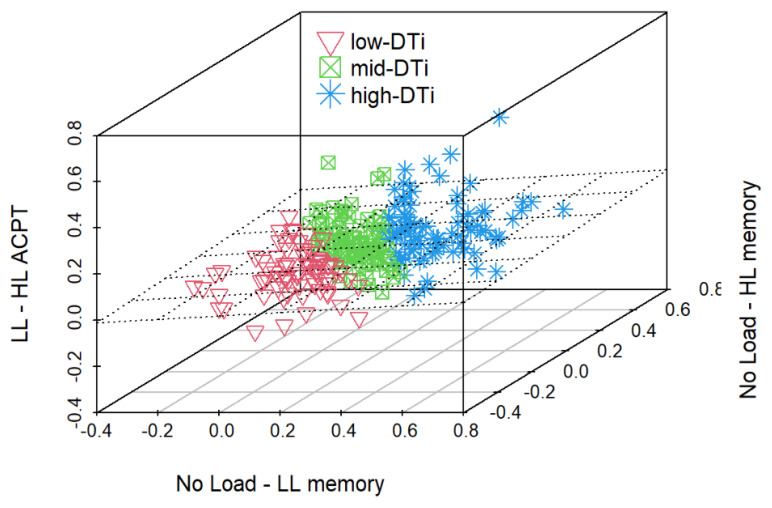
Graphical representation of participant dissimilarity calculated with a k-medoids clustering algorithm over three cost indices: the two cost indices from the memory test (No Load minus LL or HL for memory DTi) and the cost index from the ACPT (LL–HL for ACPT DTi). The cluster with low DTi includes 77 participants, the cluster with mid-DTi 88, and the cluster with high DTi 87.

The three clusters resulting from the k-medoids clustering algorithm comprised 77 participants in the low-DTi cluster, 88 in the mid-DTi cluster, and 87 in the high-DTi cluster. Descriptive statistics for Age, auto-GEMS, and Education scores for each cluster are reported in Table 7. The outcome of the clusterization is visualized in Figure 7.

**Figure 8:**
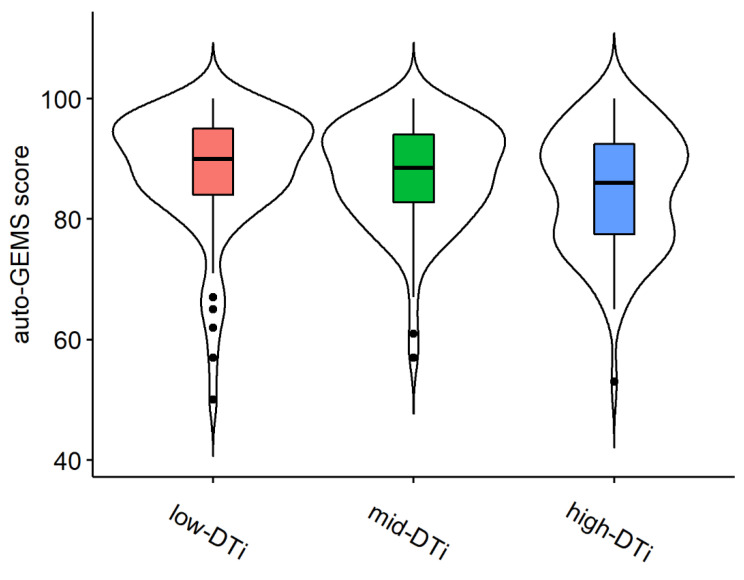
Distribution of participants’ auto-GEMS scores in the three clusters: low-DTi, mid-DTi, and high-DTi.

The rank comparison revealed no significant differences between the median ages of the three clusters of participants (Chisq = 1.945, df = 2, *p*-value = 0.378). In contrast, the differences between the auto-GEMS scores (Chisq = 8.158, df = 2, *p*-value = 0.017) and Education scores were significant (Chisq = 6.035, df = 2, *p*-value = 0.049). For the auto-GEMS scores, the high-DTi subgroup had significantly lower scores than both the mid-Dti (mean.rank.diff = 19.809, W = 4474, *p* = 0.04) and the low-DTi subgroups (mean.rank.diff = 32.057, W= 4157.5, *p* = 0.012). There was no difference between mid-Dti and low-Dti (mean.rank.diff = 12.247, W = 3761.5, *p* = 0.111) subgroups. Auto-GEMS scores for each cluster are shown in Figure 8.

For the Education score, the high-DTi subgroup had significantly lower scores than the low-DTi subgroup (mean.rank.diff = 27.831, W= 4064.5, *p* = 0.028). There was no difference between the high-DTi and mid-DTi subgroups (mean.rank.diff = 10.439, W = 4170, *p* = 0.153), and there was no difference between the mid-DTi and low-DTi subgroups (mean.rank.diff = 17.392, W = 3880.5, *p* = 0.081).

## 5. Discussion

In this study, we investigated how Dual-Task interference DTi in a cognitive–cognitive dual-task paradigm impacts performance at different ages. A cohort of healthy middle-aged and elderly participants (50–89 years) was tested with a set of online tasks. A sustained auditory attention task was performed during image memorization to study its impact on subsequent image recognition. We expected DTi to increase with increasing task demands and with the age of the participant. Surprisingly, we found that, despite a general age-related decline in mnestic and attentional performance, DTi was stable across age in both memory and sustained attention tests. Notably, the absence of the expected age-dependent increment in DTi could not be attributed to the participants’ incorrect execution of the sustained attention task. In fact, we included in the analysis only participants with performance that ensured ACPT execution in accordance with the instructions. Although no time pressure was applied to the task, we also analyzed reaction times. The reaction time results (reported in Appendix A) mirror the findings for accuracy, as they increase with cognitive load and age, but show no substantial aging–related cost increase. Therefore, the present findings are remarkably robust, as the stability of DTi across age is confirmed for both accuracy and reaction time.

Based on the evidence in this study, we can therefore argue that:

(1) There is a clear linear decline in performance in both the memory task and the sustained attention task with increasing age;
(2) There is interference between the two tasks when they are performed simultaneously, and the more demanding ACPT-AX/High Load condition results in lower performance than the ACPT-A/Low Load condition;
(3) The impact of DTi on performance is stable across ages for both tasks.

The finding that the ability to multitask does not appear to be affected by advancing age strongly contrasts with our hypothesis and with the previous literature [53]. Most, if not all, previous studies on cognitive–motor interference in MCI and dementia found a cost when multitasking which worsens with increasing age [54,55,56] and which also characterizes healthy elderlies [57,58,59,60]. However, there is one fundamental factor differentiating our study from previous ones. While multitasking experiments often focus on motor–cognitive interference, here we studied interference between two concurrent tasks that were both cognitive and that were based on different cognitive functions and sensory modalities (visual vs. auditory). By pairing an auditory sustained attention task (the ACPT) with concurrent visual object memorization, our paradigm might fit into the divided attention tasks category. According to Verhaeghen’s meta-analysis [61], specific age-related deficits emerge in tasks requiring divided attention. Surprisingly, this was not confirmed by our data, as the DTi in both tasks was stable across ages. It must be said, though, that our paradigm also differs from most other studies in which two concurrent cognitive tasks were used. These studies often use methods inspired by the psychological refractory period (PRP) literature, in which the response to the two concurrent tasks is tested on a trial-by-trial basis with a focus on response speed. Under these dual-task conditions, the performance of the elderly deteriorates more than that of the young as the load increases [62,63,64,65]. However, there are some antecedents in the literature where the differential effect of age is not observed [66,67,68]. For example, Sebastian and Mediavilla (2016) reported no effect of age or education on the amount of DTi in a box-crossing task with delayed digit recall [69]. These divergent results may reflect the effect of several experimental choices, including the cognitive functions involved, the response modality, the amount of combined load of the two tasks, and others. A recent metanalysis [70] showed that many of the individual differences in DTi (including those related to age) can be attributed to the initial ability of the participant and to the interaction with task difficulty.Age-related increases in dual-task costs are mitigated with individual load titration [70]. Although in the present study the load was not individualized, its level was particularly well-balanced to affect performance across all ages. As a result of what we learned from our previous study, we achieved an ideal level of difficulty and found no ceiling effects in the young or floor effects in the elderly [30]. The choice of task could also have other implications. Jaroslawska et al. (2021) found the amount of DTi in older participants to be strongly influenced by the nature of the task (spatial or verbal discrimination) [71]. It is, therefore, possible that it is not the case that dual-tasking ability in general declines with age, but that the often-reported increase in interference is task-dependent, as it follows the lifespan trajectory of the underlying abilities.

Is it then possible that our findings are specific to interference occurring between a memory task and a sustained attention task?

It is well known that different abilities peak and deteriorate at different ages throughout the lifespan and that the onset of age-related decline varies greatly between individuals [72,73]. On the one hand, multitasking ability might be explained by assuming a relatively independent cognitive construct [74], which might follow its developmental trajectory. On the other hand, it strongly relies on established cognitive functions such as attentional or executive resources [75], and its decline may depend on the individual timing of the different underlying skills.

The detrimental effects of load were concurrently found in two tasks with very different characteristics. This confirms the role of depletable and unspecific cognitive resources as a major determinant for performance. Such a conclusion is strongly supported by the evidence provided by brain-damaged patients whose visuospatial performance is negatively impacted to a very similar extent by a concurrent task of either visual or auditory nature [13,76]. A similar albeit less prominent pattern has been found in healthy participants by using an implicit measure such as pupil dilation [77]. In this last study the importance of “unspecific” task demands was confirmed by observing that the leading cause of performance deterioration was the difficulty in concurrently extracting the task-relevant features, rather than their number [77].

Given the structure of our memory task, during object recognition, particular emphasis was placed on familiarity rather than recollection. It is known that familiarity-based processing is mostly preserved in elderly individuals [78], while it might be somehow more severely compromised in pathological aging. Furthermore, performance in sustained attention tests is rather preserved when the task is performed with no time pressure, as in our paradigm [38]. Despite the obvious interference between the two tasks, which is also present in the young elderly, it is possible that the expected age-dependent increase did not occur because the two interfering abilities are both preserved in the old elderly. It is also possible that the fall is specific to those individuals who may have a deficit in one of the two skills involved (memory and attention) or in the ability to multitask in general. We also found that high levels of education correspond to high levels of performance in both memory and attention. Regarding the possible protective effect of education against the DTi, we found mixed evidence. On one side, there was no correlation between education and cost in the memory task. On the other side, in the ACPT the cost was lower for those with higher education.

One of the potential outcomes could have been the prioritization of either the memory or the ACPT task. On the contrary, participants did not prioritize one task over the other, as confirmed by the lack of a negative trade-off between performance in the two tasks. Rather, we found a positive—albeit not very strong—correlation between the two costs, suggesting the existence of a domain-independent ability related to multitasking. Participants who have a higher cost due to multitasking in the memory task are also those with the highest cost in the ACPT, and those who show a low cost in one task also generally have a low cost in the other. We further investigated individual differences in multitasking ability with a cluster analysis. By combining the cost information of the two tasks, we extrapolated a global interference index that was used to subdivide the sample into three subgroups, one characterized by high global interference, one with medium global interference, and one with low global interference. In line with the regression models, we did not find a significant difference between the ages of the three subgroups. This confirms the previously described pattern and further contrasts with our predictions, as it was expected that the group with high interference would also be characterized by older age. Rather, we found that the high-interference group had a significantly lower score than the other two on the Global Examination of Mental State, i.e., auto-GEMS. The high-interference group also had a lower education score than the low-interference group, but not higher than the middle-interference group, which again shows a mild protective effect of education. These results suggest that a high global interference index might be an important information to consider for identifying those “healthy” individuals who nevertheless show a lower global cognitive status than their peers.

When referring to healthy elderlies, we usually refer to undiagnosed, apparently spared elderlies. People with mild signs of cognitive decline that escape classic assessment tests might be sometimes unintentionally included in studies. Here, we effectively excluded participants who misunderstood the instructions, but also those who failed badly despite having clear instructions. If there were anyone among those tested with an undiagnosed onset of MCI, he or she was most likely excluded from the tests. It could then be possible that the age-related increase in interference only manifests itself when more subclinically impaired participants are included. The different rigor in sample selection may partly explain the differences in results between the current study and the former literature, including our previous study [30]. On the other hand, this difference in results could also depend on a likely ceiling effect in the young elderly from our previous study. While in the current study the difficulty level was effective in modulating performance in each task across all ages, in our previous research the recognition task turned out to be too easy for the young elderly, while the free recall task resulted in difficulty for the older elderly. When comparing different ages and difficulty levels, very often one of the conditions/ages presents ceiling–floor effects. Some of the previously reported interactions might be spurious, as this aspect is, to our knowledge, not systematically taken into account when interpreting data.

## 6. Conclusions

The current investigation provides novel data showing that in healthy adults, DTi is stable across age when using a cognitive–cognitive dual-task paradigm based on the interaction between a memory task and a sustained attention task. The data also show individual differences among peers in the degree of interference, reflected in differences in a global cognitive score (auto-GEMS). Although the present study did not have a clinical purpose, it is in principle possible that individual abnormal levels of DTi compared with peers indicate those subtle changes in cognitive efficiency typical of the preclinical stages of cognitive decline in the elderly.

Such cognitive measure would be an invaluable, inexpensive screening tool to determine who might be more at risk of developing full-blown cognitive decline and, ideally, who should be addressed regarding preventive cognitive stimulation or pharmacological treatments. Additionally, given the growing interest in preclinical intervention studies, measures of DTi which have been proven to be sensitive to subtle cognitive change may become important outcome measures with greater ecological validity than other laboratory measures. The social and psychological burden of dementia is such that every small step heading towards a more sensitive diagnosis/its early detection can have a very positive impact.

## Figures and Tables

**Table 1:** Accuracy (range 0–1) in the memory task, reported separately for the three Cognitive Load conditions (No Load, Low Load–LL, High Load–HL).

	Memory Task		
	No Load	Low Load	High Load
1st–3rd Qu.	0.667–0.933	0.533–0.8	0.467–0.733
Mean	0.796	0.651	0.595
SD	0.162	0.202	0.191

**Table 2:** Analysis of deviance with the type II Wald chi-square tests for memory score as a function of Cognitive Load and Age. (*** = *p*-value < 0.001).

	Chisq	Df	Pr (>Chisq)
Cognitive Load	342.867	2	<0.001 ***
Age	112.918	1	<0.001 ***
Cognitive Load X Age	5.223	2	0.073

**Table 3:** Descriptive table for the ACPT score, reported separately for ACPT-X (Low Load) and -AX (High Load).

	ACPT	
	Low Load	High Load
1st–3rd Qu.	0.933–1	0.867–1
Mean	0.972	0.919
Sd	0.055	0.097

**Table 4:** Analysis of deviance with the type II Wald chi-square tests for ACPT score as a function of load and age. (*** = *p*-value < 0.001).

	Chisq	Df	Pr (>Chisq)
Cognitive Load	95.524	1	<0.001 ***
Age	12.835	1	<0.001 ***
Cognitive Load X Age	1.603	1	0.206

**Table 5:** Descriptive table for Age, years of schooling, and Education score.

	Age	Years of Schooling	Education Score
1st–3rd Qu	55–72	8–18	89–111
Mean	63.77	12.76	100
Sd	10.745	5	13.66

**Table 6:** Analysis of deviance with the type II Wald chi-square tests for memory score as a function of Cognitive Load and Education score. (*,*** = *p*-value < 0.05 and 0.001 respectively).

	Chisq	Df	Pr (>Chisq)
Cognitive Load	340.731	2	<0.001 ***
Education Score	6.298	1	0.0121 *
Cognitive Load X Edu Score	1.603	2	0.558

**Table 7:** Descriptive table for Age, auto-GEMS score, and Education score by cluster. The three different columns encompass participants with different levels of sensitivity to dual-task interference (DTi). The low-DTi cluster (left column) includes participants whose performance suffers less from dual-tasking. By contrast, the high-DTi cluster (right column) includes participants whose performance suffers more from dual-tasking.

	Low-DTi	Mid-DTi	High-DTi
n	77	88	87
Age			
1st–3rd Qu	54–70	55.75–71.25	55.50–73.50
Mean	63	63.28	64.93
SD	10.934	10.329	11.011
**auto-GEMS**			
1st–3rd Qu	84–95	82.75–94	77.50–92.50
Mean	88.23	87.49	84.77
SD	10.159	8.519	9.846
**Education score**			
1st–3rd Qu	94–111	89–109.25	84–111
Mean	102.6	99.33	98.31
SD	12.745	12.396	15.416

**Figure A1:**
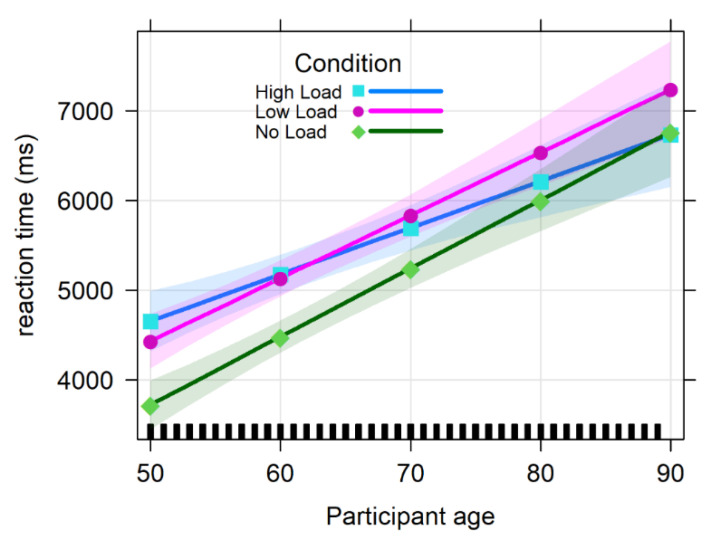
Memory reaction time as a function of Age and Cognitive load (No Load, LL, and HL). Bands represent 95% confidence intervals.

## Data Availability

The data supporting this study are available at: https://osf.io/f8c3b/?view_only=a1d1078e3b6d42b685b180e6bc8fae88 accessed on 20 June 2022.

## Appendix A

The instructions given to the participants emphasized maximum accuracy and made no specification regarding speed. During the response phase of the memory task, participants were allowed to take as much time as necessary for each of the forced choices. Regarding the ACPT, although there was no explicit time pressure, the stimuli had a rapid pace (one every 1666 ms), and therefore the participant was inclined to be fast. The analysis of reaction times was carried out using a generalized linear mixed model (GLMM) similar to that with which accuracy was analysed. Given the non-linear nature of reaction times, we chose a distribution from the inverse-Gaussian family, and we included the Cognitive Load (No Load vs. LL vs. HL) as a within-subject factor, the Age of participants as a between-subject continuous variable, and participant and Cognitive Load as a random effect in the model. For each subject, we calculated the average reaction time by dividing the total time by the number of correct answers. We then excluded from the analysis reaction times outside two standard deviations of the mean for each cognitive load condition. The analysis of deviance with the type II Wald chi-square tests for the memory score showed a significant effect of Cognitive Load (Chisq = 63.64, df = 2, *p* < 0.001), with the No Load condition resulting in a shorter reaction time compared to both the LL (LL–No Load = 637.2, se = 84.6, z = 7.529, *p* < 0.001) and HL conditions (HL–No Load = 566.6, se = 98.2, z = 5.772, *p* < 0.001). By contrast, performance in the LL condition was no different than in the HL condition (HL–LL = −70.7, se = 78.5, z = −0.900, *p* = 0.368). We also found a significant effect of Age (Chisq = 76.703, df = 1, *p* < 0.001), with the score decreasing with increasing participant age. The interaction between Cognitive Load and Age was also significant (Chisq = 9.4752, df = 2, *p* = 0.009). Looking at Figure A1, we can see that when varying the age, the No Load and LL conditions have a similar slope, in contrast to the HL condition, where the slope is smaller. In fact, although the reaction times increased as a function of load and age, the difference between the various load conditions remained the same or even decreased between young and old. This indicated that the DTi in terms of speed for the elderly was the same or even lower than for the young in the memory task.

In the memory task, we also identified a speed–accuracy trade-off characterized by a between-subjects, mild negative correlation (R = −0.19, *p* < 0.001). Participants with longer mean reaction times were also the ones with the higher mean accuracy. Concerning the ACPT, reaction times could only be measured in trials in which the target was present, and the participant responded correctly. Therefore, it was not possible to calculate a speed–accuracy trade-off within the attentional test. However, we were able to analyse speed as a function of age and cognitive load in a similar way as previously performed for the memory task. The results showed a significant effect of Cognitive Load (Chisq = 30.0808, df = 1, *p* < 0.001): (HL–LL = −128, se = 23.1, z = −5.527, *p* < 0.001). We also found a significant effect of Age (Chisq = 35.910, df = 1, *p* < 0.001), with the score decreasing with increasing participant age. The interaction between Cognitive Load and Age was not significant (Chisq = 0.016, df = 1, *p* = 0.899). For the ACPT also, the reaction times increased as a function of load and age, but the slope—and thus the difference—between the two load conditions was the same across age. This indicates that the DTi in terms of response speed for the elderly was the same as that for the young in the ACPT.
